# Antibacterial activity of epsilon-poly-l-lysine produced by *Stenotrophomonas maltophilia* HS4 and *Paenibacillus polymyxa* HS5, alone and in combination with bacteriophages

**DOI:** 10.1099/mic.0.001363

**Published:** 2023-07-21

**Authors:** Hamidreza Hagh Ranjbar, Afrouzossadat Hosseini-Abari, Seyed Mahdi Ghasemi, Zahra Hafezi Birgani

**Affiliations:** ^1^​ Department of Cell and Molecular Biology & Microbiology, Faculty of Biological Science and Technology, University of Isfahan, Isfahan, Iran; ^2^​ Department of Microbiology, Faculty of Biological Sciences and Technology, Shahid Ashrafi Esfahani University, Isfahan, Iran

**Keywords:** Antibiotic resistance, Antimicrobial Peptide, Bacteriophage, Epsilon-poly-L-lysine

## Abstract

Over the past decades, antibiotic resistance has become a major clinical problem, and searching for new therapeutic strategies seems to be necessary. Using novel natural compounds, antimicrobial peptides, and bacteriophages is the most promising solution. In this study, various cationic metabolite-producer bacteria were isolated from different soil samples. Two isolates were identified as *Stenotrophomonas maltophilia* HS4 (accession number: MW791428) and *Paenibacillus polymyxa* HS5 (accession number: MW791430) based on biochemical characteristics and phylogenetic analysis using 16S rRNA gene sequences. The cationic compound in the fermentation broth was precipitated and purified with sodium tetraphenylborate salt. The purified cationic peptide was confirmed to be epsilon-poly-l-lysine by structural and molecular analysis using High-Performance Liquid Chromatography, Sodium dodecyl-sulfate-polyacrylamide gel electrophoresis, and Fourier-transform infrared spectroscopy. The antibacterial activity of epsilon-poly-l-lysine was evaluated against *Staphylococcus aureus* ATCC 25923, *Escherichia coli* ATCC 25922, *Enterococcus faecalis* ATCC 29212, *Serratia marcescens* ATCC 13880, and *Klebsiella pneumoniae* ATCC 13883 by microdilution method. Furthermore, the antibacterial effects of purified epsilon-poly-l-lysine in combination with two long non-contractile tail bacteriophages against vancomycin-resistant *Enterococcus faecalis* and colistin-resistant *Klebsiella pneumoniae* were investigated. The results indicated great antibacterial activity of epsilon-poly-l-lysine which was produced by two novel bacteria. The epsilon-poly-l-lysine as a potent cationic antimicrobial peptide is demonstrated to possess great antimicrobial activity against pathogenic and also antibiotic-resistant bacteria.

## Introduction

Since the first antibiotic was reported in 1928 by Alexander Fleming, various antimicrobial agents have been introduced and widely used against microorganisms [1]. Within the past decades, bacteria have become resistant to antibiotics because of the use and misuse of antibiotics in medicine, animal husbandry, and agriculture [2]. Despite tremendous medical and pharmaceutical research progress, antibiotic resistance is still one of the most public health challenges [3]. Currently, using natural compounds, antimicrobial peptides, and phage therapy offer potential alternative treatments [4, 5].

Improving the efficacy of methods such as optimizing therapeutic bacteriophage and bacteriophage-derived products to overcome antibiotic resistance is necessary [6]. Specific phage-species activities exert strong selective pressure on their host population and dramatically decrease the bacterial population density. Therefore, phage therapy makes up a potential alternative approach to treat multidrug-resistant (MDR) infections [7]. However, phage-resistant mutants can appear, but applied strategies such as using phage cocktails and combining bacteriophage with other therapeutic agents, e.g. natural antimicrobial compounds, can minimize other problems [8]. Besides the use of bacteriophages, the discovery and development of novel classes of antimicrobial agents are also desired [9].

Some of the promising candidates of natural compounds with potential broad-spectrum antimicrobial activity are antimicrobial peptides (AMPs), which represent a solution to overcome antibacterial resistance [10]. AMPs in nature are produced either by ribosomal translation of mRNA or by non-ribosomal peptide synthesis. Non-ribosomal AMPs are mainly produced by bacteria, while ribosomally AMPs are genetically produced by all species of life [11]. Despite ribosomally synthesized AMPs which were recognized recently for their critical role in innate immunity, non-ribosomal peptides have been known for several decades and are used as antibiotics (e.g. polylysine, gramicidin, and polymyxins) [12].

AMPs, also known as host defence peptides, are positive charge polypeptides with potent antimicrobial activity [12]. The properties of AMPs, such as structure, size, charge, hydrophobicity, and solubility, are crucial factors for their antimicrobial activities. Cationic polypeptides with particular physicochemical characterization are impressive molecules for pharmaceutical and medical uses [13].

Even though cationic AMPs are demonstrated to possess great antimicrobial activity, rapid action, low target-based resistance, and low immunogenicity, more consideration in this area is needed to overcome high production costs, low productivity, and toxicity against eukaryotic cells. The main aim of the present study was to investigate novel cationic AMP producers with high productivity and evaluate the antibacterial activity of this cationic peptide in combination with bacteriophages.

## Methods

### Isolation and identification

Soil samples for the isolation of cationic peptide-producing bacteria were collected from different areas of Iran. The soils were suspended in normal saline and spread on a synthetic medium containing 2 g glucose, 2 g casein, 0.5 g K_2_HPO_4_, 0.01 g FeCl_3_, 0.2 g MgSO_4_, and 12 g agar per litre (pH 7.2). Cationic peptide production was screened by halo creation around colonies on agar plates containing synthetic medium with 0.02 % methylene blue (purchased from Sigma) [14].

DNA extraction was done using a DNA extraction kit (CN: EX6071, Sinnaclon, Iran). The universal primer pairs 27F (5′-AGAGTTTGATCCTGGCTCAG-3′) and 1492R (5′-AGAGTTTGATCCTGGCTCAG-3′) and the following PCR profile were used for the amplification of 16S rRNA genes: 5 min at 95 °C; 30 cycles (30 s at 95 °C, 30 s at 58 °C, 30 s at 72 °C); 10 min at 72 °C. The 16S rDNA sequences were submitted to the National Centre for Biotechnology Information (NCBI) and multiple alignments of sequences were performed to construct the phylogenetic tree. Phylogenetic analysis was accomplished by the neighbour-joining method in mega X software with 1000 bootstrap replications.

### Fermentation media and culture conditions

A single colony of different bacterial isolates with significant haloes on methylene blue-containing media was selected and inoculated into a medium containing 12.5 g glucose, 12.5 g glycerol, 10 g (NH_4_)_2_SO_4_, 5 g yeast extract, 1.6 g Na_2_HPO_4_, 0.8 g K_2_HPO_4_, 1.4 g KH_2_PO_4_, 0.5 g MgSO_4_.7H_2_O, 0.04 g ZnSO_4_.7H_2_O, and 0.03 g FeSO_4_.7H_2_O in 1000 mL water (pH 7). The ε-PL producers were cultivated for 24 h at 30 °C and 150 rpm.

### Estimation and separation of cationic peptide

The concentration of cationic peptide was estimated by an anionic dye, methyl orange [15]. Briefly, the culture broth was centrifuged at 800 RCF (Hermle Z300-K; Hermle, Wehingen, Germany), and 0.1 mL of supernatant was mixed with 1.9 mL phosphate buffer (0.1 M) and added to 2 mL methyl orange solution (0.001 M). After incubation for 45 min at 35 °C in a shaking bath, mixtures were centrifuged at 800 RCF. The absorbance of the supernatant was measured at 465 nm to estimate cationic peptide production.

To separate and purify cationic peptide from culture broth, 0.025 mL acetic acid (1 M) and 0.025 mL NaOH (0.05 M) were added to 0.5 mL bacterial supernatant. Henceforth, 0.5 mL sodium tetraphenylborate salt (NaTPB, 0.2 M) was added, shaken, and centrifuged at 14000 RCF (Daihan CF-10, DAIHAN Scientific, South Korea). To remove impurities, 1 mL acetone was added, and the supernatant was discarded gently after centrifugation at 14000 RCF. Finally, the purified cationic peptide was obtained after adding 0.05 mL of HCl (1 M) to the precipitated powder and washed with 1 mL acetone [16].

### Characterization of cationic peptide

Acidic hydrolysis of cationic peptide for analysing amino acid components was performed by heating in 6 N HCl at 110 °C for 20 h. The hydrolysed samples were finally neutralized using NaOH (4 N) solution and were passed through Agilent poroshell HPH-C18, 2.7 µm column (4.6×100 mm). The elution was conducted at a flow rate of 1.5 mL min^−1^ using 10 mM Na_2_HPO_4_, 10 mM Na_2_B_4_O_7_ (pH 8.4), and NaN_3_ as mobile phase one and acetonitrile:methanol:water (45 : 45 : 10, v:v:v) as mobile phase two [17].

The molecular weight of the purified cationic peptide was estimated using 12 % SDS-PAGE. The gel was subjected to 100 mV with 0.1 % Tris-glycine buffer (pH 8.3) for 2 h and then stained with Coomassie brilliant blue R-250 [18].

Infrared spectra of the cationic peptide were scanned at 4 cm^−1^ resolution with a frequency range between 350 and 4000 cm^−1^ using the KBr pellet method on a JASCO FTIR-6300 spectrometer (Tokyo, Japan) [19].

### Determination of antibacterial activity

The bacterial strains including *Staphylococcus aureus* ATCC 25923, *Escherichia coli* ATCC 25922, *Enterococcus faecalis* ATCC 29212, *Serratia marcescens* ATCC 13880, and *Klebsiella pneumoniae* ATCC 13883 were obtained from the University of Isfahan microbial collection.

The minimal inhibitory concentration (MIC) of the cationic peptide was evaluated quantitatively by the broth micro-dilution technique [20]. The purified cationic peptide was diluted (2 to 0.007 mg mL^−1^) in Mueller Hinton broth medium dispensed in 96-well microtitration plates. Then, 50 µL of bacterial suspension (10^6^ cells mL^−1^) was added to the wells. The microplates were incubated at 35 °C for 18 h and optical density was measured at 600 nm by a multi-mode microplate reader (HTX BioTek, Winooski, VT, USA). The minimum bactericidal concentration (MBC) was determined after microdilution by sub-culturing samples with visibly inhibited growth for an additional 24 h at 35 °C. All experiments were performed in a duplicated manner.

### Investigation of cationic peptide effects on bacteriophages

Bacteriophages used in this experiment were isolated from wastewater against *Klebsiella pneumoniae* and *Enterococcus faecalis *. The vancomycin resistant *E. faecalis* and colistin resistant *K. pneumoniae* were obtained from Nobel Medical Diagnostic Laboratory microbial collection, Isfahan, Iran. Transmission electron microscopy (TEM) was used to characterize the isolated bacteriophages [21]. To investigate cationic peptide effects on bacteriophages*,* 50 µL of sub-lethal concentrations of cationic peptide (0.25, 0.125, 0.062, and 0.031 mg mL^−1^) were mixed with 50 µL phage suspension (10^9^ PFU mL^−1^) and pre-incubated for 15 min at room temperature and then added to 100 µL of bacterial suspension (1.5×10^8^ CFU mL^−1^). Finally, the plates were incubated at 35 °C for 24 h. The antibacterial effects of the pre-incubated cationic peptide-phage mixtures were compared with the antibacterial effects of the mixtures obtained from the simultaneous mixing of the cationic peptide with the bacterial cells and the phage suspension. After 24 h*,* the absorbance of samples was measured at 600 nm by a multi-mode microplate reader (HTX BioTek, USA), and the percentage of bacterial cell viability was calculated as follows: bacterial cell viability (%) = $\left[\frac{\left(Abscontrol-Abssample\right)}{Abscontrol}\right]$ ×100. The tests were performed in duplicate.

### Statistical analysis

All experiments were conducted two times in each sample, and all results were presented as the mean value. Under the conventional acceptance of statistical significance, CI was calculated at a confidence level of 95 % using GraphPad Prism software, version 8 (GraphPad Software, USA).

## Results and discussion

### Identification of cationic peptide producing isolates

**Fig. 1.**
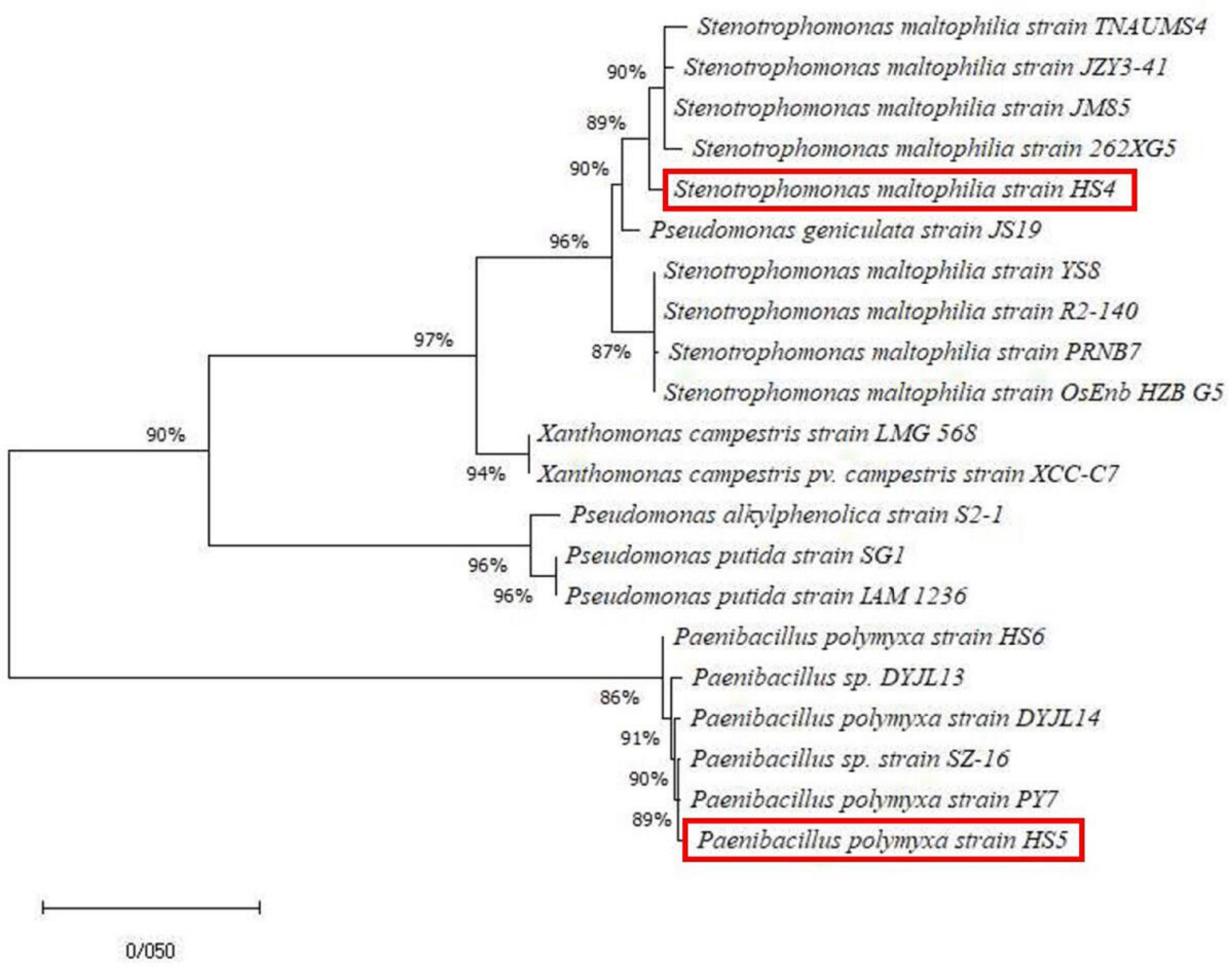
Phylogenetic tree of two novel ε-PL producers. The 16S rDNA-based dendrogram confirmed the biochemical and molecular results and the evolutionary divergence of *P. polymyxa* strain HS5 and *S. maltophilia* strain HS4. The tree was constructed using the neighbour-joining method with 1000 bootstrap replications.

The colonies that were isolated from different soil samples were cultured on the methylene blue-containing medium and those that were able to form larger halo zones were selected for cationic peptide production estimation by an anionic methyl orange dye. The two isolates which their products significantly removed methyl orange from the supernatant were selected for further analysis. Molecular analysis of the isolates showed the highest similarity to *Stenotrophomonas maltophilia* 262XG5 and *Paenibacillus polymyxa* PY7, and their 16S rDNA sequences were submitted to NCBI as *S. maltophilia* HS4 (accession number: MW791428) and *P. polymyxa* HS5 (accession number: MW791430). Phylogenetic analysis was performed using the neighbour-joining method with 1000 bootstrap replications (Fig. 1).

*P. polymyxa* and *S. maltophilia* as two crucial bacteria in the food and agriculture industries play an effective role in human and plant health by producing various metabolites [22, 23]. These bacteria, also known as plant growth-promoting bacteria (PGPB), are effective biocontrol agents by producing various AMPs. Microbial AMPs including antibiotics and antitumor agents that are produced by *P. polymyxa* are very important for human health [24, 25].

### Production and purification of cationic peptide

**Fig. 2.**
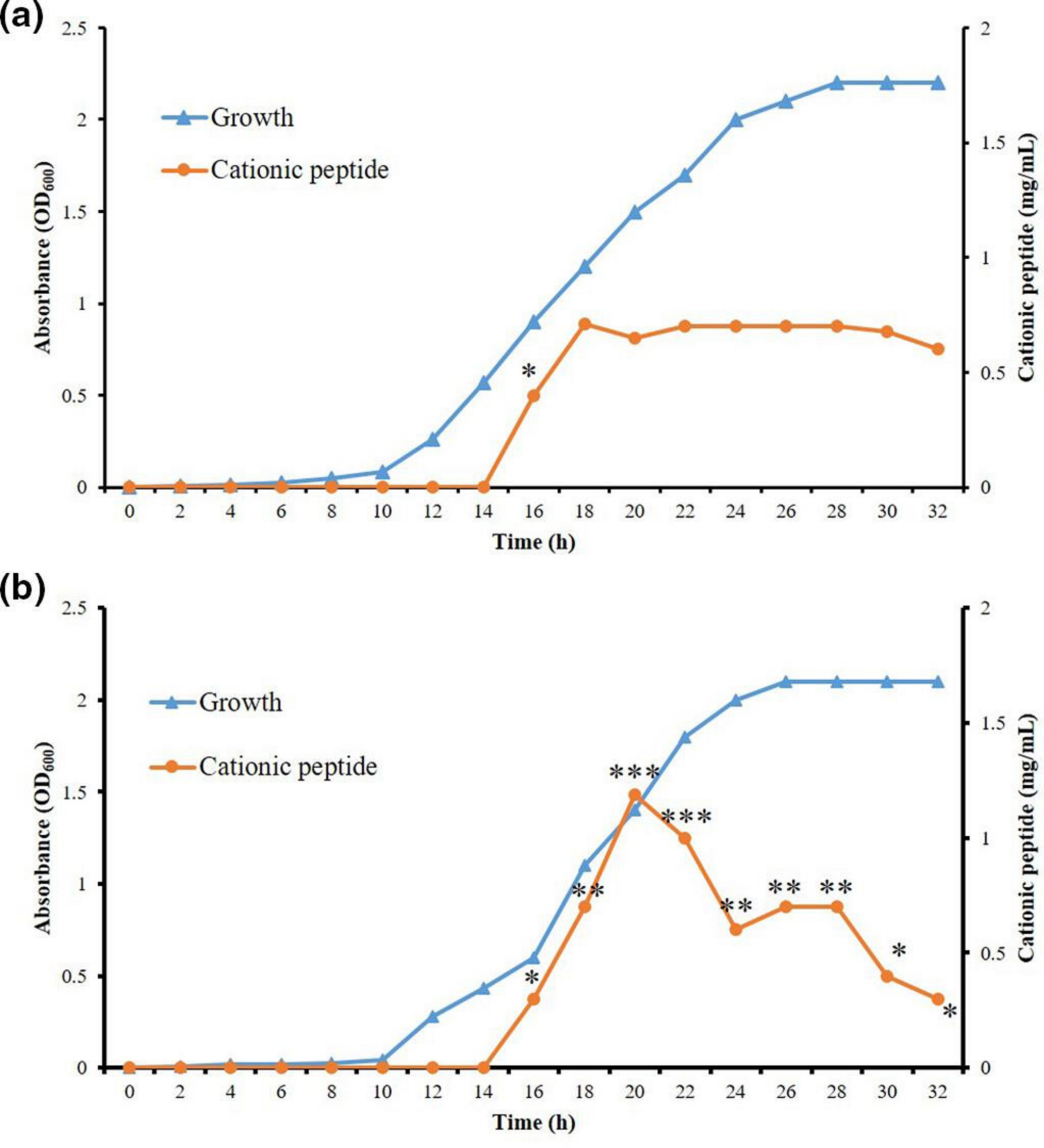
The growth curve and cationic peptide production by *S. maltophilia* HS4 (**a**) and *P. polymyxa* HS5 (**b**). The strains were grown on M3G medium at 30 °C. *, **, and *** show statistically significant differences between production rates at different times.

As shown in Fig. 2, cationic peptide production by *S. maltophilia* HS4 and *P. polymyxa* HS5 starts after 16 h of incubation and reached the highest amount after 18 and 20 h of growth, respectively. As indicated by statistical analysis in the figure using asterisks, the cationic peptide production was significantly reduced in the culture medium when the cells enter the stationary phase. This reduction occurs due to nutrient depletion (especially carbon source) and activation of degrading enzymes. Previous studies have shown that activation of ɛ-PL-degrading enzymes (Pld and PldII) plays a crucial role in the ɛ-PL reduction which is activated under the pressure of ε-PL accumulation to reduce its toxicity and maintain normal growth of the strain [26, 27].

In previous studies, many methods have been used to purify cationic peptides, but purification procedures require optimization for individual peptides and most strategies still have some problems such as poor yield, low purity, high expense, and time consumption [28]. Bankar *et al*. compared different physicochemical techniques including cation exchange chromatography, ultrafiltration, chemical precipitation, and gel permeation chromatography to purify a cationic peptide from *Streptomyces nursei* and reported that ultrafiltration and cation exchange chromatography have lower purity, while chemical precipitation and gel permeation chromatography have the highest possible purity and recovery [29]. In this study, the cationic peptide was precipitated and purified with sodium tetraphenylborate salt (NaTPB) as a chemical agent in the culture broth. The pure cationic peptide from the fermentation broth was a white powder. Precipitation by tetraphenylborate is a practical, fast, and simple approach with a high-yield product.

### Characterization of cationic peptide

**Fig. 3.**
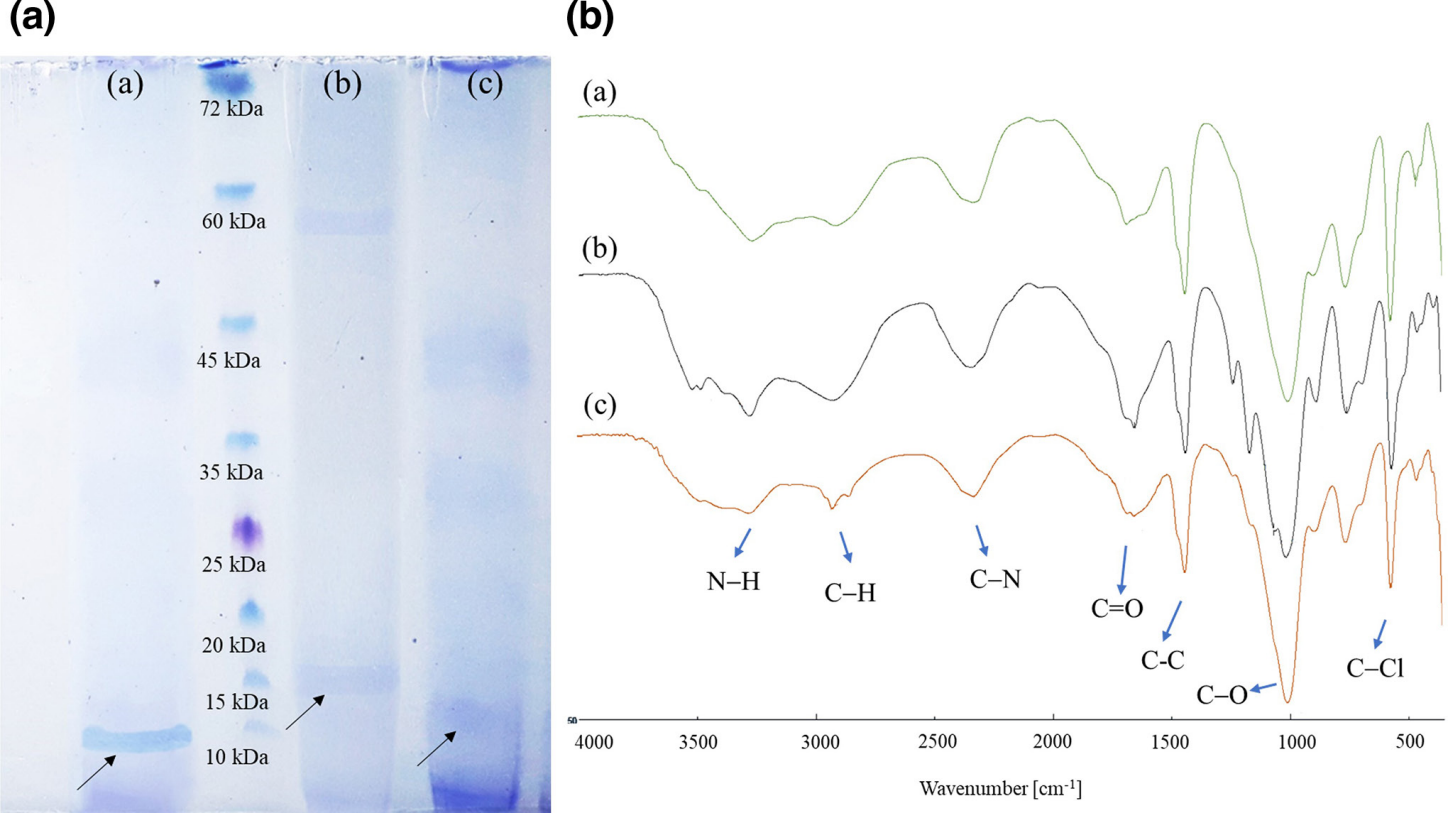
Characterization of the cationic peptide by SDS-PAGE and FTIR. Cationic peptide obtained from (a) *S. albulus,* (b) *S. maltophilia* HS4 and (c) *P. polymyxa* HS5. The arrows (↗) indicate peptide band at the expected molecular weight.

Characterization of the cationic peptide by HPLC revealed that the cationic peptide produced by *S. maltophilia* HS4 and *P. polymyxa* HS5 including 45 and 22 mM l-lysine monomers, respectively. The results suggest that the purified cationic peptide produced by the isolated strains was polylysine. In addition, the SDS-PAGE results showed that the cationic peptide from *S. maltophilia* HS4 has two bands with 60 and 12 kDa, while that of *P. polymyxa* HS5 has 10 kDa molecular weight (Fig. 3a). FT-IR analysis showed eight H signals (Fig. 3b). Some strong peaks at 550–900 cm^−1^ show C-Cl or H-Cl bonds, and the peaks at 1000–1200 cm^−1^ are related to C-O bonds. IR spectra of the carbonyl group have a strong peak at 1640–1750 cm^−1^. Strong stretching peaks at the 2900–3000 cm^−1^ range are related to C-H and C-R. Middle peaks at 3100–3400 cm^−1^ illustrate neutral amide (NH group) and side-chain amine (NH_2_ groups). Protonated side-chain NH_3_^+^ group showed middle peaks at 2500–3100 cm^−1^. Finally, the product was confirmed to be epsilon-poly-l-lysine (ε-PL) consistent with a similar pattern of ε-PL produced by *S. albulus* as a common ε-PL producer.

Epsilon-Poly-l-Lysine (ε-PL) as one of the well-known AMPs is a natural cationic polypeptide formed by at least ten lysine residues linked by peptide bonds between the amine and carboxyl group [30]. Since its first discovery in 1977 by Shima and Sakai using *S. albulus *, the structure, synthesis, and wide application range of *ε*-PL have been thoroughly researched in recent years [31]. Recently, the production of *ε*-PL has been increased by various methods, including the use of a pH shock strategy, a two-stage pH control strategy, and the addition of different compounds such as astaxanthin, citrate, and others [32–34].

Particular properties alongside various advantages provide *ε*-PL to be used in modifying drugs and improving their compatibility and affinity. Remarkable properties, such as biodegradability, heat stability, and water solubility along with a wide range of antimicrobial properties encourage the use of *ε*-PL in various industrial and pharmaceutical fields, including agriculture, food, chemistry, and biomedical [35]. In recent years, ε-PL has become a great candidate to use as suture or dressing material when conjugated with other biomaterials such as cellulose, hyaluronic acid, and chitosan due to its exhibited antimicrobial and antibiofilm ability without inducing an inflammatory response [36–40].

In this study, *S. maltophilia* HS4 and *P. polymyxa* HS5 were introduced as two novel ε-PL producers with a maximum yield of 0.711 and 1.19 g L^−1^, respectively. Production of ε-PL in strains with a better productivity rate, more products in less time**,** in comparison with *Streptomyces* can increase the production efficiency of this valuable peptide. Also, the synthesis of AMPs such as *ε*-PL with a better productivity rate not only increases the yield of production but also drives production costs down compared to chemical synthesis. The yield of ε-PL in other non-optimized production strategies and non-artificial strains is given in Table 1.

**Table 1.** The comparison of ɛ-PL production in the isolated strains and the other wild type ɛ-PL producers

Bacteria	Max. yield (g L^−1^)	Period (h)	Reference
* S. maltophilia* HS4	0.711	18	This study
* P. polymyxa* HS5	1.19	20	This study
* S. albulus* NBRC 14147	1.2	50	[51]
* S. albulus* W-156	2.3	120	[52]
* Streptomyces griseofuscus* H1	0.8	84	[53]
*Streptomyces gniseoaurantiacus*	0.2	120	[54]
* Bacillus subtilis* SDNS	0.076	14	[55]

### Antibacterial activity of ε-PL

The antibacterial activity of ε-PL was shown in Table 2. According to previous studies, ε-PL as a natural antimicrobial peptide can inactivate microorganisms including bacteria, yeast, and viruses [41]. Being cationic allows ε-PL to interact with negatively charged molecules and cell surfaces such as bacterial membranes, viral components, and fungal cell walls [42, 43].

**Table 2.** Minimal inhibitory and bactericidal concentration of ɛ-PL produced by *S. maltophilia* HS4, *P. polymyxa* HS5 and *S. albulus* (control) against five standard bacteria

Microorganism	ε-PL from * S. maltophilia* HS4		ε-PL from * P. polymyxa* HS5		ε-PL from * S. albulus *	
	MIC (mg mL^−1^)	MBC (mg mL^−1^)	MIC (mg mL^−1^)	MBC (mg mL^−1^)	MIC (mg mL^−1^)	MBC (mg mL^−1^)
* S. marcescens* ATCC 13880	0.5	1	1	2	0.5	1
* E. coli* ATCC 25922	1	1	–	–	0.5	1
*K. pneumoniae ATCC 13883*	0.5	1	0.5	1	0.5	1
* S. aureus* ATCC 25923	0.125	0.125	0.062	0.125	0.062	0.062
* E. faecalis* ATCC 29212	0.125	0.25	0.25	0.5	0.25	0.5

Liu *et al*. have shown that electrostatic interaction and the peptide backbone structure of ε-PL, which is affected by pH, could induce different antibacterial activity. Also, they revealed that the antibacterial property of ε-PL is correlated to changes in pH during separation and purification [44]. The source of electrostatic interaction is the side chain amino group, and the protonated amino group under acidic conditions has a strong positive charge to promote the adsorption of ε-PL on the bacterial membrane but as approaches the isoelectric point, the protonation decreases until reaches zero at the isoelectric point [42]. It seems that by reducing the binding affinity of ε-PL to negatively charged compounds in the membrane, it accumulates on the surface of the membrane and disturbs the balance of membrane charges which finally leads to bacterial cell destruction.

The production of ε-PL in *S. maltophilia* HS4 and *P. polymyxa* HS5 is controlled by non-ribosomal peptide systems that support the production of variable peptide sizes with different antibacterial activity. The antimicrobial activity of ε-PL depends on the molecular weight of the peptide which increases directly with increasing molecular weight. Thus, a different number of l-lysine residues produced by *S. maltophilia* HS4, *P. polymyxa* HS5, and *S. albulus* might be the reason for different MIC and MBC values obtained against the tested bacteria. According to previous studies, ɛ-PL requires a minimum of ten l-lysine residues to exhibit antibacterial activity [45].

In this study, the susceptibility of the microorganisms to ε-PL showed that the Gram-positive bacteria are more susceptible in comparison with Gram-negatives. Positioning between the extracellular medium and the cytosol makes the cell membrane a primary target for damage caused by AMPs such as ε-PL that inhibit microorganisms by the disturbance of integrity and function of the cell membrane [46]. Based on the previous study, ε-PL induced structural change of peptidoglycan and other molecules and caused the cell wall to become more fragile. The permeability of the cell membrane was also increased by ε-PL [47]. The less susceptibility of Gram-negative bacteria against this cationic AMP may be because of the difference in the cell wall structure. In addition, the results of Bo *et al*. showed that ε-PL led to the inhibition of intracellular carbon metabolism [48].

In the study of Liu *et al*. which was conducted on *B. subtilis *, *E. coli,* and *S. aureus* in different pH, it was found that the most antimicrobial effect is observed in the pH range of 5 to 9 and the effect of ε-PL on Gram-positive bacteria is greater than Gram-negative bacteria at this range [44]. In this study, we adjusted pH to eight for separating and seven for investigating the antibacterial activity, and in confirmation of Liu *et al*., we concluded that the ε-PL produced by *S. maltopilia* HS4 and *P. polymyxa* HS5 is more effective against Gram-positive bacteria compared to Gram-negatives. One of the debatable theories for the difference in the results of the antibacterial effects of ε-PL on Gram-positive and Gram-negative bacteria in previous studies can be the lack of pH consideration.

Different concentrations of ε-PL have different effects on the cell membrane and metabolic pathways. Tan *et al*. showed that intracellular metabolites including amino acids, lipids, carbohydrates, and alcohols have significantly changed in treating with high concentration ε-PL. In addition, the inhibitory effect of ε-PL on glycolysis, Embden–Meyerhof–Parnas (EMP), and TCA cycle was observed because of a self-protection mechanism [41]. Finally, the studies suggested that the ε-PL could cause cell disruption through various effects on cell metabolism and cell surface.

### Antimicrobial effects of ε-PL in combination with bacteriophages

**Fig. 4.**
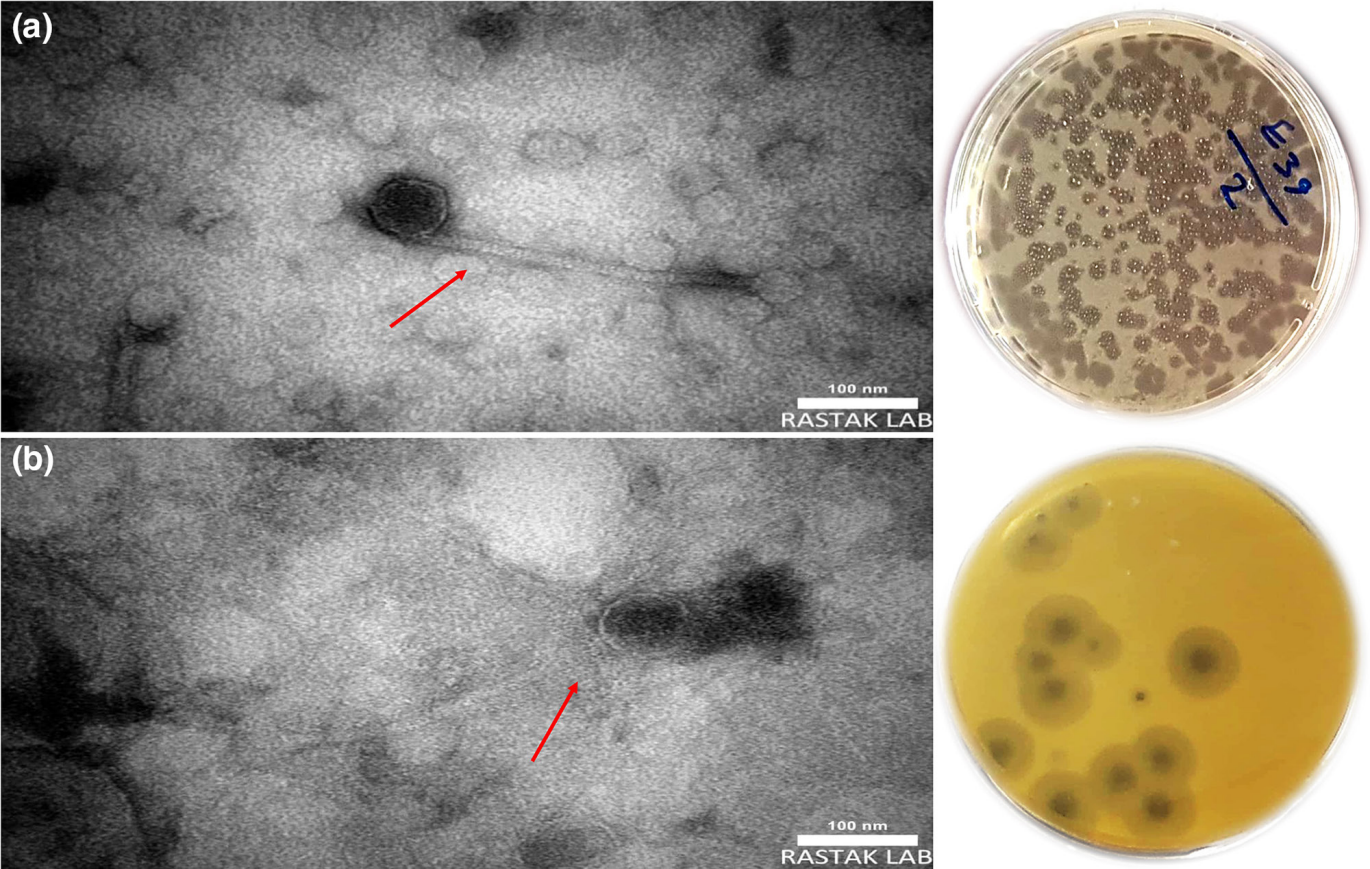
Plaque morphology and TEM images of (a) bacteriophage against vancomycin-resistant *Enterococcus faecalis* and (b) bacteriophage against colistin-resistant *Klebsiella pneumoniae *.

Bacteriophages used in this experiment were isolated from wastewater against *K. pneumoniae* and *E. faecalis *. The TEM images revealed that both bacteriophages have an icosahedral head and a long non-contractile tail, suggesting that they belong to the class *Caudoviricetes* (formerly classified as the order *Caudovirales* and the family *Siphoviridae*). The plaque appearance which formed a clear zone on the BHI medium at 37 °C suggests that they are lytic phages (Fig. 4).

To investigate cationic peptide effects on bacteriophages*,* sub-lethal concentrations of cationic peptide were mixed with bacteriophage suspensions and pre-incubated for 15 min at room temperature, and then added to the bacterial suspensions. Although AMPs and bacteriophages can be effective alternatives to antibiotics on their own, improving their effectiveness is necessary to combat multidrug-resistant bacteria [49]. In this study, we chose *K. pneumoniae* and *E. faecalis* as two important pathogenic bacteria to investigate the antimicrobial effects of ε-PL alone and in combination with bacteriophages. *K. pneumoniae* and *E. faecalis *, which primarily affect debilitated and immunocompromised patients, are quickly developing multidrug-resistant strains and commonly pose a serious threat to the patients because of an increased fatality rate due to the reduced effectiveness of therapy options.

**Fig. 5.**
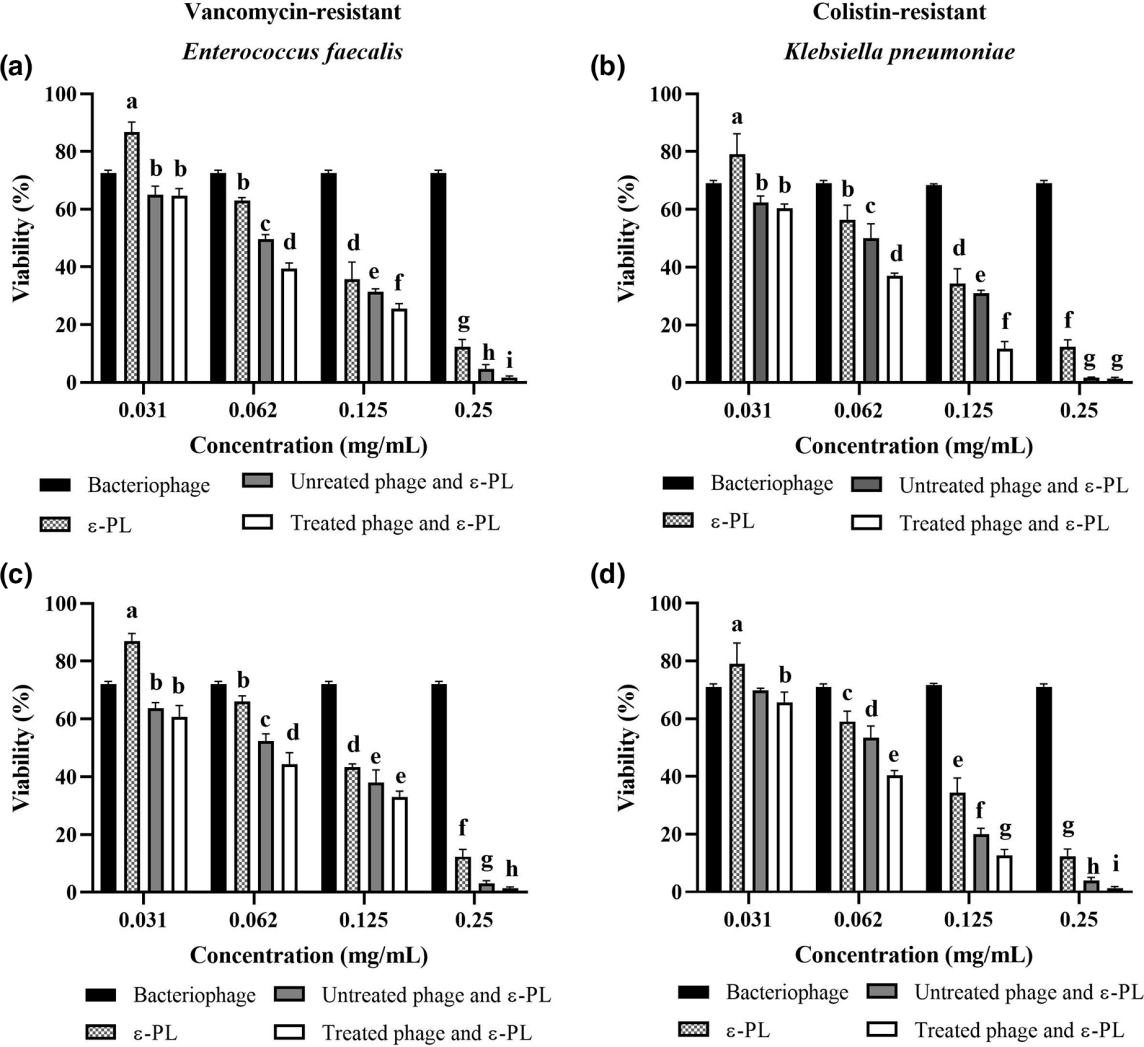
Antibacterial Activity of ε-PL alone and combined with bacteriophages against *K. pneumoniae* and *E. faecalis *. (**a and b**) ε-PL obtained from *S. maltophilia* HS4 and (**c and d**) ε-PL obtained from *P. polymyxa* HS5. Different letters indicate statistically significant differences between different concentration of treatments in each diagram (*P*<0.05) according to Duncan’s test. Treated: pretreated phage and ε-PL for 15 min +bacteria, Untreated: phage + ε-PL+bacteria.

As shown in Fig. 5, the combination of the long non-contractile-tailed bacteriophages and ε-PL at all concentrations significantly reduced bacterial viability. Different letters in each diagram indicate statistically significant differences at a confidence level of 95 % between reduced cell viability treated by ε-PL, bacteriophages, pre-incubated and non-incubated bacteriophages/ε-PL. Combined ε-PL with phages reduced bacterial viability by over 95 % at 0.25 mg mL^−1^, while cell viability was reduced by approximately 30 and 84% when phages and ε-PL were used alone, respectively. Also, statistically significant differences between pre-incubated and non-incubated bacteriophages and ε-PL produced by two novel strains were observed at all concentrations except 0.031 mg mL^−1^. The ε-PL produced by novel strains at 0.031 showed low antibacterial activity with a maximum 22 % viability reduction of *K. pneumoniae *. Pre-incubation of bacteriophage and 0.125 mg mL^−1^ of ε-PL produced by *S. maltophilia* HS4 decreased *K. pneumoniae* viability about three-fold higher than unincubated phage and 1.5-fold more than the same condition with ε-PL produced by *P. polymyxa* HS5. Concerning the control cultures, there was no significant difference between bacteriophage activity against *E. faecalis* and *K. pneumoniae *.

The results showed that the use of bacteriophage is significantly more effective than the ε-PL at 0.031 mg mL^−1^, but with increasing concentration, ε-PL is more effective compared to bacteriophages. In general, it can be concluded that ε-PL and bacteriophages can be effective for reducing cell viability alone, but the statistical analysis (indicated by different letters in each graph in Fig. 5) showed that the use of ε-PL and bacteriophage together, regardless of pre-treatment or not, reduced cell viability more in comparison with using them alone. Also, the results showed that the pre-treatment of phages and ε-PL for 15 min significantly reduced the cell viability compared to their non-treated in most of the investigated concentrations. It seems that ε-PL (cationic) interacts with the anionic groups of the phage proteins, immobilizes on the phage surface, and then antibacterial activity increases. The rates of damage were increased in correlation to the concentration.

Limited studies are available on the interaction between ε-PL and bacteriophages. A previous study by Shima *et al*. investigated the inactivation of bacteriophages by ε-PL produced by *S. albulus *. The result of their study indicated that phage morphology and environmental condition (e.g. incubation temperature and pH) are two crucial factors in ε-PL and bacteriophage interactions [50]. In addition, their study revealed that the ε-PL inhibits contractile-tailed *E. coli* T4 phage and long non-contractile-tailed *E. coli* T5 phage while the results of this study indicated that the ε-PL not only has no inhibited effects on phages but also increased their efficacy against host bacteria.

The main aim of the present study was to explore cationic peptide-producing bacteria and evaluate the antibacterial potency of this peptide. Production of ε-PL and its use in medicine and the pharmaceutical industry have great economic and health importance. In this study*, S. maltophilia* HS4 and *P. polymyxa* HS5 were introduced as two novel ε-PL producing bacteria. According to the results obtained in this study, the ε-PL produced by the two novel producers is a potent cationic compound with great antibacterial properties, with IC50 0.119 and 0.132 mg mL^−1^ for *S. maltophilia* HS4 and *P. polymyxa* HS5, respectively. In addition, the effects of this cationic peptide with bacteriophages offers a potential method for their combined use to combat microbial resistance. Furthermore, the result of this study suggested that pre-incubation of phages and ε-PL could improve antibacterial effects in comparison with monotherapy which can be considered for further studies regarding the combination use of AMPs and bacteriophages.
